# Pigmentation and Degradative Activity of TiO_2_ on Polyethylene Films Using Masterbatches Fabricated Using Variable-Frequency Ultrasound-Assisted Melt-Extrusion

**DOI:** 10.3390/ma13173855

**Published:** 2020-09-01

**Authors:** Christian J. Cabello-Alvarado, Zoe V. Quiñones-Jurado, Víctor J. Cruz-Delgado, Carlos A. Avila-Orta

**Affiliations:** 1 Consejo Nacional de Ciencia y Tecnologia, Centro de Investigación y de Innovación del Estado de Tlaxcala, Tlaxcala C.P. 90000, Mexico; christian.cabello@conacyt.mx; 2 Departamento de Materiales Avanzados, Centro de Investigación en Química Aplicada, Saltillo, Coahuila C.P. 25294, Mexico; 3 Facultad de Ciencias Químicas, Universidad Juárez del Estado de Durango, Durango C.P. 34120, Mexico; 4 Departamento de Procesos de Transformación, Centro de Investigación en Química Aplicada, Saltillo, Coahuila C.P. 25294, Mexico; victor.cruz@ciqa.edu.mx

**Keywords:** ultrasound-assisted, melt-extrusion, white films, photodegradation, TiO_2_ pigment

## Abstract

Ultrasound-assisted melt-extrusion method (USME) is a high-quality process used to produce polymeric compounds with an adequate homogeneous dispersion. This study evaluates white-color films of linear low-density polyethylene (LLDPE) prepared using TiO_2_ masterbatch obtained by ultrasound-assisted melt-extrusion at variable frequencies (USME-VF). LLDPE with three different melt-flow indices (2, 20 and 50 g/10 min) were used as the polymer matrix. The films were obtained from the dilution of masterbatches of LLDPE (melt-flow index = 2) at a concentration of 7 wt% TiO_2_. The morphology, pigmentation, TiO_2_ reactivity, and the mechanical stability of the films were assessed. The masterbatch compounds were evaluated by melt-flow index (MFI) and scanning electron microscopy (SEM). The contrast ratio, yellowness index and mechanical properties of films were also measured. The properties of whiteness and elongation at break improved in the films prepared using masterbatches with higher dispersion. Though the reactivity of the TiO_2_ particles increased during accelerated aging, it did not affect the elongation to rupture. The yellowness index was moderately affected in films that included TiO_2_ particles processed using USME-VF.

## 1. Introduction

In order to achieve numerous specifications of packaging functionality, additives and inorganic fillers in polymers have been incorporated. Properties such as gas barrier, antistatic, antimicrobial, optical, among others have been reported to achieve good appearance [1,2,3]. In packaging, coating appearance and technology can be decisive for the acceptance of products [4]. Even the value and quality performance of products can be primarily attributed to color characteristics [5].

Optical properties such as color, brightness and hiding power typically depend on pigment chemistry, concentration and dispersion in a matrix or substrate. These factors are decisive in the interaction of pigments with wavelength of visible light [6]. Titanium dioxide (TiO_2_)—with a rutile crystal structure—is considered the most competent white pigment because its pigmentation strength is maximized by its high refractive index (2.74) [7] and by the lack of absorption of visible light [8].

The hiding power—or opacity imparted by the TiO_2_—is the capacity of pigment to hide the visual aspect of a substrate, and it can be increased by (1) avoiding the agglomeration of these particles and (2) promoting the interaction of TiO_2_ particles with wavelength at 560 nm (the center of the visible spectrum). The excellent whiteness using TiO_2_ in polymers can be achieved by maximizing the high refractive index along the three axes of rutile crystal [9] and the light scattering coefficient related to the diameter of the TiO_2_ particle, which is in the range of 180 to 300 nm [10].

The energy required to optimize the dispersion of TiO_2_ pigment in polymers is related to the attraction forces between particles and affinity between components [11]. Dispersion of TiO_2_ into polymers is commonly obtained during melt-extrusion (ME) and depends on the mix processes, equipment (such as extruder type single, twin-screw extruder, co-rotating or counter-rotating, intermeshing or non-intermeshing) and screw design [12]. Even the melt-flow index of the polymer matrix is another factor that plays an important role to enable the transmission of dispersion energy [13].

Additionally, it is still possible to gain further optimization of TiO_2_ dispersion using ultrasonic radiation in the extrusion process [14]. This process is designated as ultrasound-assisted melt-extrusion (USME).

Ultrasonic radiation produces turbulent flow conditions in a melted or liquid substance due to alternate compression and rarefaction phases of sound waves [15]. Therefore, USME process can increase the shear stresses in the molten polymer and promote a better homogenization of pigment in polymeric compounds. The increase in energy by the USME method has shown an improvement in the deagglomeration of particles in polymer composites and the ability to fracture agglomerates when they are tightly bound as aggregates [16,17].

Through the years, the ultrasonic application on fixed frequency has been dominating [14,18,19]. This work studies the dispersion of TiO_2_ pigment in LLDPE by melt-extrusion, applying ultrasonic waves at variable frequency (USME-VF). Ultrasonic waves at variable frequency are a nonconventional ultrasound method [20]. This latter implies the vibration of polymer chains in a dynamic scanning frequency range. While ultrasonic waves at a fixed frequency only can interact with polymer chains of a specific length or molecular weight [21].

This study aims to analyze the dispersion effect of TiO_2_ pigment in the preparation of white masterbatch by implementing the USME-VF and studying the influence of TiO_2_ particles after masterbatch dilution on both pigmentation and photodegradation on linear low-density polyethylene (LLDPE) polymer composites.

## 2. Materials and Methods

### 2.1. Materials

LLDPE was provided by A. Shulman Company of Mexico, with three different melt-flow index (MFI) 2, 20 and 50 g/10 min, which will be named PE2, PE20 and PE50 from now on. TiO_2_ pigment was purchased from the Chemours Co., (Altamira, Mexico) with a particle size of approximately 200 nm.

### 2.2. Preparation of Masterbatch LLDPE/TiO_2_ and White Films

Masterbatch of LLDPE/TiO_2_, with 10, 20 and 30 wt% of TiO_2_ (called MB10, MB20 and MB30, respectively) using different LLDPEs with melt-flow indices of 2, 20 and 50 g/10 min (PE2, PE2 and PE50) were generated in a twin-screw extruder from Thermo Scientific model Prism TSE-24MC (Stone, UK), with screw diameter of 24 mm, L/D ratio 40:1, temperature profile of 190 °C and rotational speed of 100 rpm. The screw configuration involved two zones of high shredding. A specially designed adapter for ultrasound treatment was fixed at the exit of the extruder for the application of ultrasound waves, as previously reported [20], where the temperature in ultrasound system was the same that in extruder die. A homemade ultrasonic generator (Centro de Investigación en Química Aplicada, Saltillo, Mexico) produced the ultrasonic waves between a range of 15–50 kHz and with a power of 750 W. For the addition of polymer pellets, a Brabender volumetric feeder (Brabender-Technologie, Duisburg, Germany) coupled to the extruder was used at an extrusion rate from 3.8 to 4.2 kg/h. A side gravimetric feeder type Movacolor (Sneek, Netherlands) was set at 60% of their capacity and the TiO_2_ powder feeder was set at 10, 20 and 30% (Table 1). Finally, concentrated masterbatches were used to obtain white films on a KTS-100 cast film extrusion system (Windsor Machines, Ahmedabad, India) at a temperature of 200 °C. The thickness of the LLDPE films was 50 micrometers (ASTM D6998). The masterbatch compounds were blended with LLDPE to fix an amount of TiO_2_ in the film of 7 wt% (Table 2).

### 2.3. Characterization Techniques

The prepared masterbatch’s melt-flow index was evaluated using an extrusion plastometer in accordance with the ASTM D 1238 standard method. For this study, a Dynisco (Dynisco, Heilbronn, Germany) Melt Indexer was used at 220 °C, with a weight of 2.16 kg. Five measurements were made, and the average value is reported.

For Dispersion Measurement, the sample surfaces were coated with a layer of gold with a thickness of 8–10 nm. A JOEL Field Emission scanning electron microscope model JSM-7401 F was used. The microscope acceleration voltage was 3.0 kV using the LEI secondary electrons detector generator (JEOL, Tokyo, Japan).

The opacity percent developed by the pigment on the films was evaluated following the ASTM-D-2805 method, using a Chroma CS-5 spectrophotometer (Chroma Technology Corp, Vermont, VT, USA). The standard associates the reflected light when the films are placed over a black surface. Three measurements were done, and the average value is reported.

Chromaticity of YI was obtained from color coordinates measured by using a CM-3600d Spectrophotometer Konica Minolta Sensing (Osaka, Japan), with an observation angle of 10°, using an Illuminant D65 standard. YI per ASTM Method E313 is calculated as follows: 100(1.3013X−1.1498Z)/Y, where X, Y and Z are the CIE Tristimulus values.

Photodegradation activity of TiO_2_ Pigment Particles under the accelerated aging method was performed using the Weathering Tester (QUV) equipment, model QUV spray UV40, from QPanel Lab Products (Baltimore, USA) under the following conditions; radiation UV at 0.89 W/m^2^/nm at 340 nm, setting an exposure cycle at 60 °C lasting for 8 h and an additional period of condensation cycle with a spray of deionized water at 50 °C lasting for 4 h. Monitoring time under aging exposition was of 100, 200, 300, 400, 500 h. Additionally, changes in mechanical properties of films were assessed by retention of elongation at break with an Extensometer Model Long Travel, Instron Model 4467 (Instron, Jackson, MS, USA), according to the standard methods ASTM D638 and ASTM D618.

## 3. Results and Discussion

### 3.1. Masterbatch Concentrates and Raw Materials

#### 3.1.1. Melt Flow Index (MFI) of Polymers and Masterbatches Fabricated with and without USME-VF

The MFI value for TiO_2_ masterbatch concentrates obtained by ME and USME-VF was analyzed and compared to the raw LLDPE, respectively. Table 3 shows that the MFI was maintained close to unprocessed LLDPE. For the PE2 matrix, a slight increase at MFI value was presented after processing in ME and USME-VF. While the masterbatches prepared with the PE20 matrix, the MFI increased with both the TiO_2_ content and the USME-VF process. In masterbatch samples composed of PE50 by ME without ultrasound treatment, the flow rate decreased, which may be due to the size of the agglomerates of the TiO_2_ particles and the poor distribution of the agglomerates in the polymer matrix. However, when the ultrasound energy was applied, an increase in MFI was observed, possibly because the particles could exhibit greater surface area and performing as lubricants in the polymer matrix [22]. However, the increasing of MFI also could be related to the degradation of polymeric matrix. Polymer degradation can be due to long ultrasonic treatment time, high temperature, high ultrasound power and a short distance of the polymeric matrix to the tip of the ultrasonic probe, causing a decrease in molecular weight, in viscosity, viscoelastic modules and relaxation time, as shown in the literature [23,24]. Other studies indicate that polymeric compounds with inorganic pigments processed under high temperature begin to dehydrate and dehydroxylate and therefore, the moisture can attack and degrade the polymer, even at low loading percentages [25]. The above suggest that values of MFI of LLDPE can be associated with polymer degradation, and this affect could be more pronounced for samples processed by USME-VF.

#### 3.1.2. SEM Study of the Dispersion Developed by ME and USME-VF

**Figure 1:**
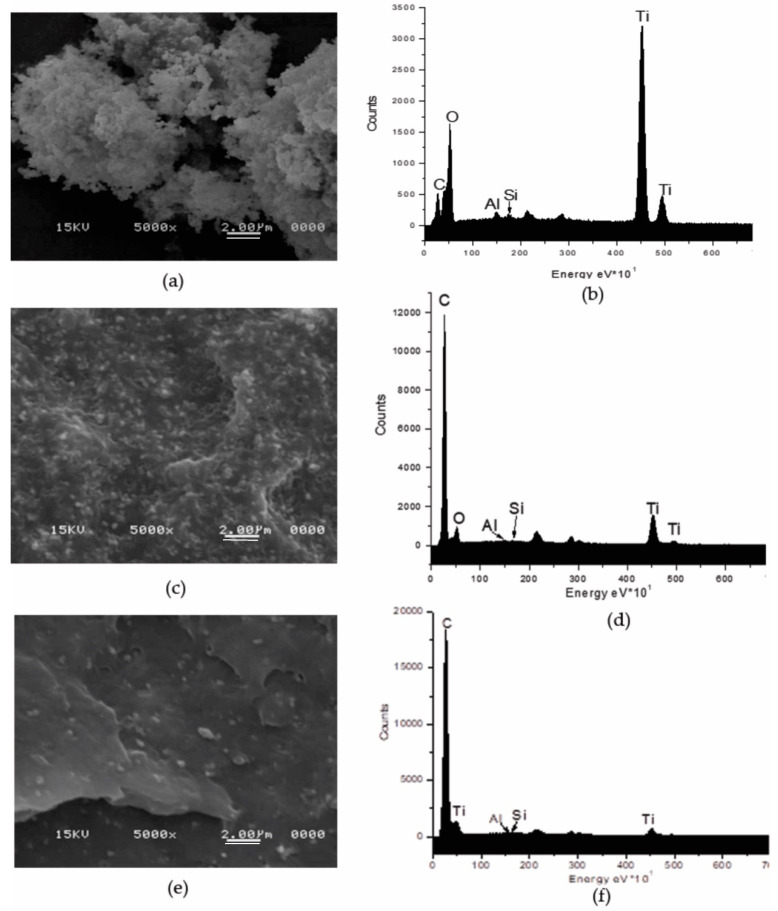
SEM images at 5000× of the TiO_2_ particles, its dispersion and EDS spectra. (**a**,**b**) As-received TiO_2_ powder; (**c**,**d**) dispersed in PE by extrusion (MB30.PE2); (**e**,**f**) dispersed in PE by USME-VF (MB30.PE2. USME-VF).

The dispersion of TiO_2_ was analyzed by scanning electron microscopy. Figure 1a shows the SEM image at 5000× for the as-received TiO_2_ powder. This image displays hemispherical particles forming agglomerates. EDX spectrum corroborated the elemental composition of TiO_2_, besides of titanium and oxygen, these particles contain Al, Si and C (Figure 1b). The Al and the Si are elements usually present in the form of oxides or hydroxides to passivate the surface of TiO_2_ and the C to enhance the compatibility of the nonpolar polyolefin [26,27,28,29]. The proportion of each detected element in terms of the mass fraction was 9.53, 33.13, 0.62, 0.41 and 56.3 wt% for the C, O, Al, Si, Ti, respectively.

Figure 1c,e correspond to the images of the composites manufactured with and without ultrasound, containing 30 wt% TiO_2_ particles in a PE matrix with MFI = 2. Figure 1c is the micrograph of TiO_2_ incorporation in PE without applying ultrasound treatment. This image shows that dispersion obtained with the traditional melt-extrusion process (ME) can reduce the size of the agglomerates, presenting clusters of particles from one micron and some isolated particles are also observed. However, the use of USME-VF results in a better dispersion of TiO_2_ particles in the PE2 polymeric matrix (Figure 1e). By comparing the signals of the EDX spectra of compound obtained by ME (Figure 1d) and USME-VF (Figure 1f), it is observed that the carbon signal for the polymer matrix is intensified. This effect is more pronounced for the masterbatch prepared by USME-VF method (Figure 1f). This may be due to the fact that TiO_2_ particle content becomes less detectable since the particles are better distributed in the polymer matrix. This result confirmed that the homogenization in the masterbatch treated with variable frequency ultrasound was promoted, as reported by Bernhardt et al. [30], who performed experiments with ultrasonic energy at low power percentages (3 watts) and different frequencies had found an advantage on the dispersion of dyes and fillers in thermoplastic materials.

#### 3.1.3. SEM Study of the TiO_2_ Dispersion Developed by USME-VF Method in PE Matrix of Different Rheology

**Figure 2:**
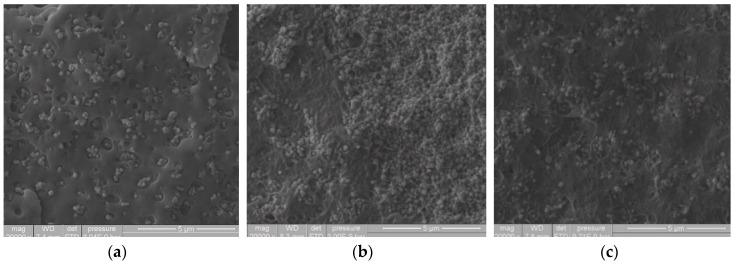
SEM images at 20,000× of the dispersed pigment in masterbatch compounds fabricated by USME-VF, based on 30 wt% of TiO_2_ and PE matrix of different rheology. (**a**) MFI = 2; (**b**) MFI = 20; (**c**) MFI = 50.

The effect of ultrasonic treatment at a variable frequency range of 15 to 50 kHz on polymeric matrices with different MFIs was analyzed. Figure 2 shows the masterbatch microstructure at 30 wt% of the pigment (TiO_2_), using different PE matrices (MFI 2, 20 and 50, respectively). This figure show how the compound morphology changed depending on the polymer MFI. In Figure 2a) it is shown that the compound with the polymeric matrix of the lowest fluidity index (MFI = 2) had marks or gaps close to the interface between the pigment and the polymeric matrix; this may be due to the high viscosity of this polymer. On the other hand, the morphology of the compound with MFI 20 (Figure 2b), presented a more significant aspect of roughness in the polymer matrix than the one with an MFI = 2. This type of morphology has been observed in compounds of PP/TiO_2_ [31]. However, the effect of the ultrasound treatment on the compound with MFI 50 (Figure 2c), showed less evidence of marks or craters in the polymer matrix and in the polymer-pigment interface, this may be because there is a greater relaxation of polymer chains, which are less entanglement due to the lower molecular weight [32]. By comparing the achieved dispersion in the masterbatch concentrates, it is observed that the dispersion of TiO_2_ in the LLDPE matrix with MFI 2 showed a more excellent uniformity in the distribution of the particles. In the polymer matrix with MFI 50, the particles tend to bind. It can be considered that the polymer matrix with a higher fluidity index may have more significant movement. Thus, the particles tend to re-agglomerate; this behavior has been observed when obtaining nanocomposites of Nylon 6 and Cu nanoparticles by ultrasound-assisted extrusion; this phenomenon could be occurring for the high fluidity index of LLDPE [33].

### 3.2. Films Pigmented with Masterbatch Concentrates Based at TiO_2_ in LLDPE Matrix Produced by USME-VF Method

#### 3.2.1. Effect of TiO_2_ Dispersion on the Film Pigmentation

This study focuses on the characterization on films developed from the PE2 masterbatch concentrates, since these high viscosity compounds (MFI 2) showed the best dispersion of particles.

**Figure 3:**
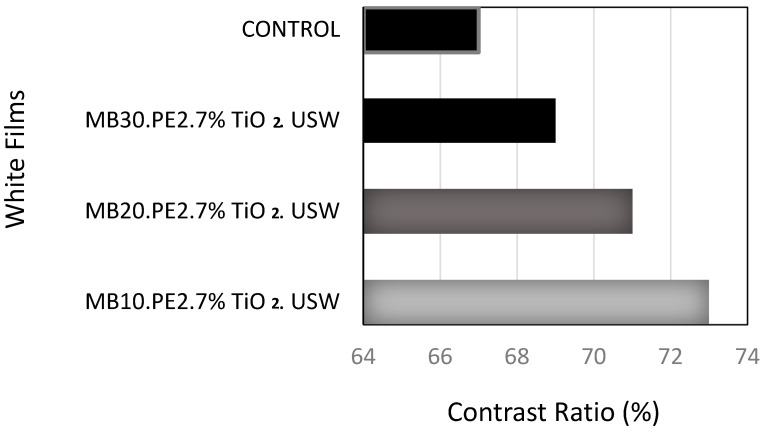
Comparison of the contrast ratio of the control (MB10.PE2.7% TiO_2_ control) film versus white LDPE films, related to the optic effect achieved by dispersion of 7 wt% of TiO_2_, using masterbatch compounds concentrated by ultrasound-assisted extrusion method at 10, 20 and 30 wt% of TiO_2_, blended in PE matrix of MFI 2.

Figure 3 shows the capacity of the covering power of pigmented films with 7 wt% of TiO_2_ at 50 microns of thickness. The pigmentation achieved was dependent on the concentration of the masterbatches produced by ultrasonic assisted extrusion process. As control film, the film produced from the concentrate without ultrasound treatment was used.

When the white films were contrasted against a black substrate, the pigmentation of TiO_2_ was better for samples prepared using the masterbatch concentrates obtained by the USME-VF method. In addition, it was confirmed that the film pigmentation at the settled concentration of TiO_2_ at 7 wt% was dependent on the concentration of pigment particles in the masterbatch of MFI 2. As observed in Figure 3, the masterbatch of lower TiO_2_ concentration the best capacity for covering power of the films. The capability of pigmentation of the masterbatches was as follow MB10.PE2. USME-VF > MB20.PE2. USME-VF > MB30.PE2. USME-VF, it could be related to the lower agglomeration or crowding of TiO_2_ particles at low concentrations.

**Figure 4:**
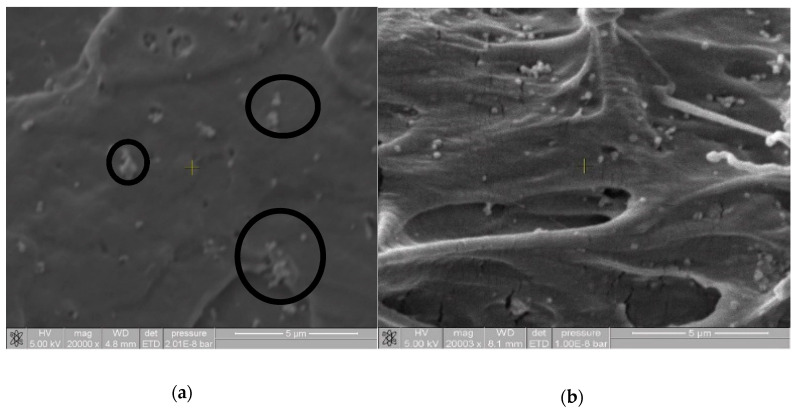
SEM images at 20,000× of pigment dispersion (7 wt% TiO_2_) in films prepared using the masterbatch MB10.PE2. USME-VF. (**a**) MB10.PE2.7% TiO_2_ control; (**b**) MB10.PE2.7% TiO_2_.USW.

Investigations carried out using TiO_2_ as a pigment have indicated that the covering power increases with dispersion of TiO_2_ in the polymer matrix, due to the different refractive indices of the air and the polymeric compound, which helps give an increase in the contrast range [34]. Figure 4 shows the SEM images of films prepared using the MB10.PE2 masterbatch resulting of ME and USME-VF, where (a) is MB10.PE2.7% TiO_2_ control and (b) MB10.PE2.7% TiO_2_. USW. These images show that the control sample has different areas with the agglomeration of the pigment due to the lumps of the TiO_2_ particles. On the contrary, in Figure 4b, a homogeneous distribution of TiO_2_ particles can be seen in the polymer matrix; this may be due to the deagglomerating effect by ultrasound energy.

#### 3.2.2. Photocatalytic Degradation of LLDPE Films Containing TiO_2_ Pigment Processed with or without Ultrasound

Since TiO_2_ is a semiconductor material, it can absorb ultraviolet radiation and promote the electrons displacement and, consequently, the formation of holes or electron vacancies, whose presence can increase the surface photocatalytic activity in TiO_2_ particles [35].

**Figure 5:**
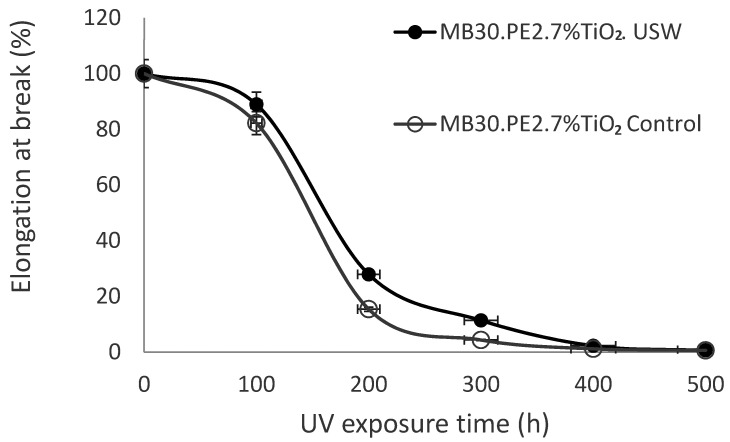
Effect of USME-VF process on TiO_2_ prodegradant effect by indirect measure of elongation at break in white films with TiO_2_ at 7 wt%.

The mechanical property of elongation to rupture in the films was analyzed after exposing them to an accelerated UV aging treatment, to identify whether the ultrasound-assisted extrusion process influences the prodegradant effect of TiO_2_. Results of the mechanical resistance on films conformed with masterbatch MB30.PE2, prepared by both ultrasound and conventional extrusion (control) are shown in Figure 5, which displays that the mechanical stability was not influenced by the photocatalytic change of TiO_2_ undergoing ultrasound waves. Even conversely, after accelerated aging treatment, the rupture elongation of the films prepared from the masterbatch with USME-VF had a better performance than with the use of masterbatch obtained by ME, due precisely by improved dispersion using the ultrasound in USME-VF. This observation is in accord with the literature, which refers that mechanical properties of tensile stress, elongation at break, Young’s modulus, and toughness in HDPE and PP polymers could be increased by an appropriate dispersion of TiO_2_ using different processes of conventional extrusion, emulsion and in situ polymerization [36,37,38,39,40].

Yellowness index study was carried out to identify the color change of the films after aging treatment. The prodegradant effect of the pristine TiO_2_ particles and of these after USME-VF process were evaluated. The yellowness appearance in the PE material may be observed if it suffers an oxidative degradation, causing of breaking of PE chains and adversely of detriment in its mechanical properties [39]. In this study, PE oxidative aging is subject to increasing of photocatalytic activity in TiO_2_ particles. Therefore, it is essential to verify if the TiO_2_ reactivity could be increased by applying the ultrasonic waves in a dynamic scanning frequency range.

**Figure 6:**
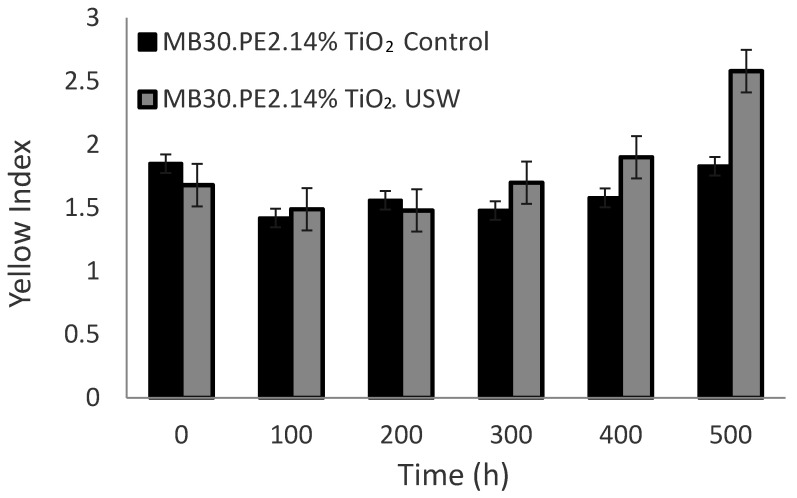
Effect of standard extrusion vs. ultrasound-extrusion on the yellowness index of films pigmented with TiO_2_ at 7 wt%.

The use of ultrasound in the extrusion process could contribute to deagglomerate TiO_2_ particles in the polymer matrix, and therefore to increase the whiteness of the compound, this behavior is observed in Figure 6, where at zero hours, the films obtained from the concentrates with ultrasound (MB30.PE2. USW) shown a less yellowness index than the control film. However, when exposed to UV radiation, it was observed that TiO_2_ particles dispersed with ultrasound caused a slight increase in the yellowness index [41].

After undergoing an accelerated aging treatment, the films at 200 h, the yellowness index begins to be more noticeable in materials processed using USME-VF than in materials processed without ultrasound. At 400 h, both films containing TiO_2_ processed with and without ultrasound exhibit a yellowness index close to 1.5%. After 500 h of treatment, the material processed with ultrasound again exceeds the value of the yellowness index compared to the material with the TiO_2_ control, reaching a value of 2.3%.

## 4. Conclusions

In this study, masterbatch concentrates of TiO_2_ and LLDPE were prepared by the ultrasound-assisted melt-extrusion method, in variable frequency mode.

The traditional extrusion process could reduce the size of the agglomerates, presenting clusters of particles from one micron. Nevertheless, better dispersion of TiO_2_ particles in the LLDPE polymeric matrix by the processing of ultrasound-assisted extrusion was obtained. It was noticed that the polymeric matrix of lower fluidity (MFI 2) in the masterbatch resulted in a better pigmentation. Masterbatches of MFI 2 showed a better capacity of pigmentation and covering power in the films when reducing the agglomeration or crowding of TiO_2_ particles. This result was achieved with the masterbatch at low concentrations of TiO_2_ (MB10.PE2. USW). The deagglomeration process using variable ultrasound frequency increased the photocatalytic activity of the TiO_2_ particles of the films exposed to accelerate aging. However, a better mechanical behavior of the elongation at rupture of films was promoted by achieving the deagglomeration of TiO_2_ particles. The increase in the particle photocatalytic activity was not decisive for degrading the films, and it was found that only after undergoing accelerated aging, the films showed a slight increase in the yellowness index.

## Figures and Tables

**Table 1:** Composition and processing conditions of all the masterbatch samples.

Sample without Ultrasonic Treatment	MFI of LLDPE (g/10 min)	TiO_2_ (wt%)	Samples with Ultrasonic Treatment	MFI of LLDPE	TiO_2_ (wt%)
MB10.PE2	2	10	MB10.PE2. USME-VF	2	10
MB20.PE2	2	20	MB20.PE2. USME-VF	2	20
MB30.PE2	2	30	MB30.PE2. USME-VF	2	30
MB10.PE20	20	10	MB10.PE20. USME-VF	20	10
MB20.PE20	20	20	MB20.PE20. USME-VF	20	20
MB30.PE20	20	30	MB30.PE20. USME-VF	20	30
MB10.PE50	50	10	MB10.PE50. USME-VF	50	10
MB20.PE50	50	20	MB20.PE50. USME-VF	50	20
MB30.PE50	50	30	MB30.PE50. USME-VF	50	30

USME-VF—ultrasound-assisted melt-extrusion method variable frequency; MFI—Melt flow index; LLDPE—linear low-density polyethylene.

**Table 2:** Composition and processing conditions of all the films.

Sample	MFI of LLDPE	TiO_2_ (wt%)	Source Master Batch
MB10.PE2.7% TiO_2_ control	2	7	MB10.PE2
MB10.PE2.7% TiO_2_ control	2	7	MB20.PE2
MB10.PE2.7% TiO_2_ control	2	7	MB30.PE2
MB10.PE2.7% TiO_2_. USW	2	7	MB10.PE2. USME-VF
MB20.PE2.7% TiO_2_. USW	2	7	MB20.PE2. USME-VF
MB30.PE2.7% TiO_2_. USW	2	7	MB30.PE2. USME-VF

**Table 3:** Melt flow index for polymers and masterbatches, evaluated at 220 °C/2.16 kg.

Sample	MFI	Sample	MFI	Sample	MFI
Resins					
PE2	1.74	PE20	19.14	PE50	52.27
ME					
MB10.PE2	2.04	MB10.PE20	17.75	MB10.PE50	47.81
MB20. PE2	2.01	MB20. PE20	18.12	MB20. PE50	48.21
MB30.PE2	2.05	MB30.PE20	20.69	MB30.PE50	47.62
USME					
MB10.PE2. USME-VF	2.12	MB10.PE20. USME-VF	19.08	MB10.PE50. USME-VF	47.04
MB20. PE2. USME-VF	1.92	MB20. PE20. USME-VF	21.17	MB20. PE50. USME-VF	53.76
MB30.PE2. USME-VF	2.07	MB30.PE20. USME-VF	22.63	MB30.PE50. USME-VF	57.32
